# Ablation guided by STAR‐mapping in addition to pulmonary vein isolation is superior to pulmonary vein isolation alone or in combination with CFAE/linear ablation for persistent AF

**DOI:** 10.1111/jce.14856

**Published:** 2021-01-09

**Authors:** Shohreh Honarbakhsh, Richard J. Schilling, Rui Providencia, Gurpreet Dhillon, Omotomilola Bajomo, Emily Keating, Malcolm Finlay, Ross J. Hunter

**Affiliations:** ^1^ Barts Heart Centre Barts Health NHS Trust London UK; ^2^ Queen Mary University of London

**Keywords:** atrial fibrillation, catheter ablation, mapping, pulmonary vein isolation

## Abstract

**Introduction:**

The optimal ablation approach for persistent atrial fibrillation (AF) remains unclear.

**Methods and Results:**

Objective was to compare the long‐term rates of freedom from AF/AT in patients that underwent STAR mapping guided ablation against outcomes of patients undergoing conventional ablation procedures.

Patients undergoing ablation for persistent AF as part of the Stochastic Trajectory Analysis of Ranked signals (STAR) mapping study were included. Outcomes following 'pulmonary vein isolation (PVI) plus STAR mapping guided ablation (STAR mapping cohort) were compared to patients undergoing PVI alone ablation during the same time period and also a propensity‐matched cohort undergoing PVI plus the addition of complex fractionated electrogram (CFAE) and/or linear ablation (“conventional ablation”). Rates of procedural AF termination and freedom from AF/AT during follow‐up were compared.

Sixty‐five patients were included in both the STAR cohort and propensity matched conventional ablation cohort. AF termination rates were significantly higher in the STAR cohort (51/65, 78.5%) than conventional ablation cohort (10/65, 15.4%) and PVI alone ablation cohort (13/50, 26.0%; STAR cohort vs. other 2 cohorts both *p* < .001). There was no significant difference in procedure time between the three cohorts. During ≥20 months follow‐up a lower proportion of patients had AF/AT recurrence in the STAR cohort (20.0%) compared with the conventional ablation cohort (50.8%) or the PVI alone ablation cohort (50.0%; both *p* < .05 compared to STAR cohort).

**Conclusions:**

Outcomes of PVI plus STAR mapping guided ablation was superior to PVI alone or in combination with linear/CFAE ablation. A multicenter randomized controlled trial is planned to confirm these findings.

## 1 INTRODUCTION

There is ongoing lack of consensus in regard to the ablation strategy that should be adopted in persistent atrial fibrillation (AF). The STAR AF2 trial demonstrated no significant difference in freedom from AF/atrial tachycardia (AT) when comparing pulmonary vein isolation (PVI) versus PVI plus additional ablation including complex fractionated electrograms (CFAEs) or linear ablation.^1^ This has led many to conclude that PVI alone should be adopted for persistent AF. However, with success rates reported at approximately 50% following the first procedure this is still not optimal. More recent studies have adopted an ablation strategy involving targeting localized drivers in addition to PVI, however, these have shown conflicting results.^2^, ^3^, ^4^, ^5^, ^6^ The most recent RE‐AFFIRM trial demonstrated no impact on outcomes when comparing a PVI only approach against PVI plus localized driver ablation guided by the FIRMap system.^7^

Stochastic Trajectory Analysis of Ranked Signals (STAR) method is a novel‐mapping method for identifying localized sources that potentially play a mechanistic role in maintaining AF. The principle of STAR mapping is to use data on multiple individual wavefront trajectories to identify atrial regions which most often precede activation of neighboring areas with the aim of identifying intermittent drivers.^8^, ^9^ The STAR mapping method has been validated in vitro using optical mapping of calcium transit in spontaneous fibrillating HL1 cells and in human studies mapping atrial paced beats and ATs.^9^ The STAR mapping method has been used prospectively to guide ablation of localized drivers in addition to PVI using both global mapping with whole‐chamber basket catheters and sequential mapping with multipolar catheters and has been associated with a high freedom from AF/AT during long‐term follow‐up.^8^, ^10^, ^11^, ^12^ It has also been shown that AF drivers (AFD) identified when using baskets catheters to obtain the unipolar recordings are also in a majority of cases also identified when using pulmonary vein (PV) mapping catheters.^10^ The aim of this study was to compare the long‐term rates of freedom from AF/AT in patients that underwent persistent AF ablation guided by the STAR mapping method against outcomes of patients undergoing conventional ablation procedures. We compared outcomes in the STAR guided ablation cohort to (1) all patients that underwent PVI alone ablation (the “PVI alone” cohort) during the same time‐period, and (2) a propensity matched cohort of patients that underwent PVI and additional ablation in the form of lines and/or CFAEs over the same time‐period (the “conventional ablation” cohort).

## 2 METHODS

### 2.1 STAR mapping principals

**Figure 1:**
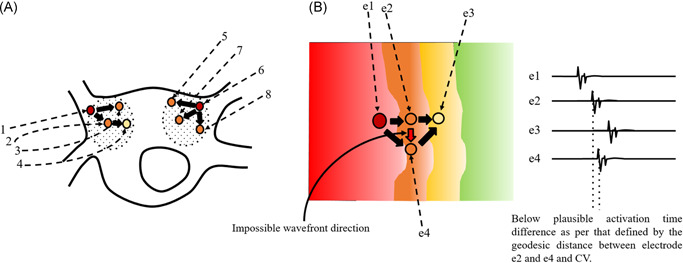
Demonstrates the principles of the STAR mapping method. (A) Shows electrodes (small circles) paired within a pre‐defined geodesic distance (highlighted by the dashed circles). The color of the circles represents the proportion of time an electrode is leading in comparison with the electrodes it is paired with. In this illustration the darker the color the greater proportion of time the electrode is leading. The arrows demonstrate the direction of the wavefront propagation. Electrode 1 is leading its pairs (Electrodes 2, 3, and 4) and Electrode 6 is leading its pairs (Electrode 5, 7, and 8). (B) Shows two wavefront trajectories. In one wavefront trajectory the wavefront propagates from Electrode e1 to e2 and then e3. In the other wavefront trajectory the wavefront propagates from Electrode e1 to e4 and then e3. The STAR mapping method excludes the wavefront trajectory of e2 to e4 as the activation time difference between these two electrodes falls below the plausible activation time difference defined by CV and the geodesic distance between the two electrodes. CV, conduction velocity; STAR, Stochastic Trajectory Analysis of Ranked signals

The STAR mapping method has been described in detail in previously published work.^8^, ^9^ In brief, the principle of STAR mapping is to use data from multiple individual wavefront trajectories to identify regions of the atrium that most often precede activation of neighboring areas. By gathering data from many hundreds of activations, a statistical model can be formed. This permits regions of the atrium to be ranked according to the amount of time that activations precede those of adjacent regions (Figure 1A,B). Electrodes are paired using pre‐defined geodesic distances, the activation times are then compared between the electrodes in all pairs using all wavefront trajectories to establish a leading site and thereby the direction of wavefront propagation (Figure 1A,B). For leading sites to be classified as AFD they are required to lead at least 75% of the time. Unipolar activation times were taken at the peak negative dv/dt. To exclude implausible wavefront trajectories the STAR mapping method filters wavefront activations based on plausible activation time differences established using geodesic distance and pre‐defined conduction velocities (CVs; Figure 1A,B). To avoid annotations on noise or fractionated electrograms, signals are filtered using the refractory period.

### 2.2 STAR mapping cohort

#### 2.2.1 Patient selection

Patients with persistent AF were included (continuous AF duration < 24 months and no previous AF ablation). All patients provided informed consent for their study participation. Ethical approval was granted by the UK National Research Ethics System (16/LO/1379). The study was prospectively registered on clinicaltrials. gov (NCT02950844). Patients included in this study have been included in our previous work.^8^, ^12^

#### 2.2.2 STAR mapping ablation

All procedures were performed using a three‐dimensional (3D) mapping system (CARTO Biosense Webster; Rhythmia, Boston Scientific, and EnSite Precision system, Abbott). All patients underwent PVI with wide area circumferential ablation (WACA) using an irrigated radiofrequency ablation catheter. Twenty minutes following PVI further ablation in the left atrium (LA) was guided by the STAR maps created using imported unipolar electrogram signals, chamber geometry, and catheter electrode location data that were processed by a Matlab custom written script (Matlab 2017b, MathWorks) written by the authors (SH). The STAR maps were created using unipolar recordings that were obtained post‐PVI either using whole‐chamber basket catheters (Constellation, Boston Scientific or FIRMap Abbott) or PV mapping catheters (PentaRay catheter with CARTO, IntellaMap Orion with Rhythmia and Advisor HD Grid with EnSite Precision System). Prospective studies have shown that both of these mapping modalities can effectively identify AFD.^8^, ^10^, ^11^ When using the basket catheters a minimum of two recordings were taken in different positions if required to achieve optimal LA coverage. With PV mapping catheters a minimum of 10 recordings were taken to ensure optimal LA coverage. The STAR maps created in Matlab were used to identify AFD that were projected onto a replica of the geometry which had been created using the 3D mapping system, which allows the location of the AFD identified on the Matlab STAR map to be tagged on the geometry on the 3D mapping system. All AFD on a STAR map were targeted in order of ranked priority whereby sites that were leading 100% of the time were targeted first followed by those leading 90% of the time, and so forth. If multiple AFD with the same ranked priority were identified it was at the operator's discretion in what order to ablate the AFD.

Initial mapping and ablation was performed in the LA, and right atrium (RA) mapping was only performed if AFD in the RA was considered likely (coronary sinus [CS] activation predominantly proximal to distal and fastest cycle length (CL) at the LA septum).

During ablation of AFD, a lesion was delivered at the center of the driver site with further ablation surrounding the initial lesion in a cluster, avoiding the creation of linear lesions so as not to affect any AF mechanisms in this way. Ablation at driver sites was delivered with a contact force of 5–40 *g*, with a power of 30–40 W (30 W posteriorly and 40 W elsewhere). Ablation at a driver site was stopped if: a total of 5 min of ablation had been performed at a driver site including consolidating ablation lesions, or no signal remained at the ablation site, or a study‐defined ablation response had been achieved. A study‐defined ablation response was either AF termination or CL slowing of more than or equal to 30 ms. If AF terminated before other AFD had been ablated, these sites were not empirically targeted. Beyond targeting AFD no other empirical ablation was allowed including the creation of lines. If AF organized into an AT this was mapped and ablated during the procedure. DC cardioversion was performed at the end of the procedure if AF did not terminate following ablation of all identified AFD.

#### 2.2.3 Follow‐up

All patients in the STAR guided ablation cohort underwent clinical follow‐up at 3, 6, and 12 months, with 48‐h ambulatory Holter monitoring at 6 and 12 months. Patients were followed up 6 monthly thereafter. A 3‐month “blanking period” was observed, with all medication including antiarrhythmic drugs continued during this time. Clinical success was defined as freedom from AF/AT lasting more than 30 s off antiarrhythmic drugs subsequent to the 3 month blanking period after a single procedure, as per consensus recommendations.^13^

### 2.3 PVI alone and conventional ablation cohorts

To identify the propensity‐matched “conventional ablation” cohort all patients that underwent persistent AF ablation during the same time‐period as the STAR mapping patients were identified from the prospective institution registry. All patients that had undergone a previous AF ablation were excluded leaving de‐novo persistent AF ablations. Patients that underwent cryoablation were excluded to only include patients that underwent radiofrequency ablation, so as to keep the method of PVI consistent with the STAR mapping cohort. Patients that only underwent PVI using radiofrequency catheter ablation formed the “PVI alone ablation” cohort and were excluded from the propensity matched cohort.

The remaining cohort consisted of patients that all underwent PVI plus CFAE or linear ablation or both with the aim of terminating to sinus rhythm. This cohort will be referred to “conventional ablation” cohort. These patients were propensity matched to the STAR mapping cohort.

The aim of the study was not to propensity match the PVI alone ablation cohort to the STAR mapping patients. The number of consecutive patients undergoing PVI alone ablation during the same time period as the STAR mapping patients were smaller making propensity matching unfeasible.

Patients in the propensity matched cohort and those in the PVI alone cohort all underwent a minimum follow‐up of 12 months with further follow‐up at the physician's discretion. All patients had a documented 12‐lead ECG performed at 3, 6, and 12 months follow‐up and Holter monitoring was performed in accordance to patient's symptoms and clinicians discretion.

Long‐term clinical outcomes were compared between the STAR mapping guided ablation cohort and both the PVI alone cohort and the conventional ablation cohort. As a procedural end point, rates of AF termination were compared between cohorts. As with the STAR mapping cohorts, clinical success was defined as freedom from AF/AT lasting more than 30 s off antiarrhythmic drugs subsequent to the 3 month blanking period after a single procedure, as per consensus recommendations.^13^ Procedural metrics were also compared between groups, including rates of the electrophysiologic end point of AF termination during ablation. All catheter ablation procedures in the three groups were consultant led with similar years of experience.

### 2.4 Statistical analysis

Statistical analyses were performed using SPSS (IBM SPSS Statistics, Version 24 IBM Corp.). Continuous variables are displayed as mean ± standard deviation (*SD*) or median (interquartile range). Categorical variables are presented as a number and percentage. The *χ* ^2^ was used for the comparison of nominal variables. The Student's *t*‐test, or its nonparametric equivalent, Mann–Whitney U test when appropriate was used for comparison of continuous variables. A *p*‐value of less than .05 was deemed significant.

A propensity score was obtained for all eligible participants undergoing persistent AF ablation through binary logistic regression: ablation strategy (STAR mapping or conventional ablation) was the binary outcome and all baseline variables (Table S1) were used as covariates for estimating a probability (the propensity score). Then, probabilities in the STAR mapping cohort were matched 1:1 to the closest conventional ablation ablation cohort patient fulfilling inclusion criteria using the nearest neighbor matching approach. The propensity score was matched to five decimals whenever possible. If this was not possible, we subsequently attempted 4, 3, and then 2 decimal matching.

Comparisons between the STAR mapping and conventional ablation cohort were performed. Based on Stuart,^14^ analyses were performed using the cohorts as a whole, rather than using the individual matched pairs.

Kaplan–Meier curves were traced for comparing survival free from AF/AT during follow‐up among the three treatment cohorts. For the purpose of time to event analysis only time to first event was considered, the patients were censored after their first event.

## 3 RESULTS

### 3.1 STAR mapping versus propensity matched cohort

#### 3.1.1 Baseline characteristics

Sixty‐five patients were included in the STAR mapping study. Thirty‐five of these were performed using a basket catheter and 30 utilized a PV mapping catheter. Outcomes for these patients have been published at earlier timepoints.

**Figure 2:**
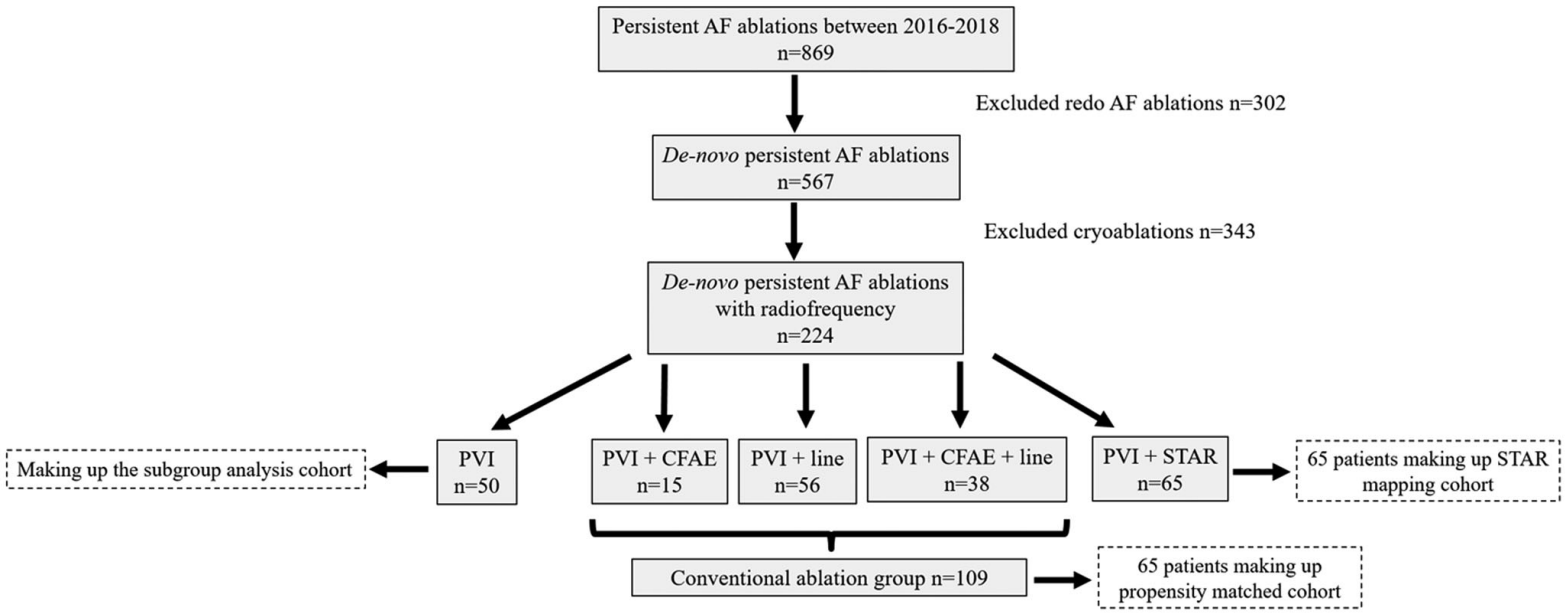
A flow diagram that breaks down how the propensity matched cohort was established in this study. AF, atrial fibrillation; CFAE, complex fractionated electrograms; PVI, pulmonary vein isolation; STAR, Stochastic Trajectory Analysis of Ranked signals

These patients were propensity matched to a conventional ablation cohort. Figure 2 demonstrates the establishment of the conventional ablation cohort. Table 1 compares the baseline characteristics of these two cohorts. There was no significant difference in the use of antiarrhythmic drugs (46/65 vs. 44/65; *p* = .85), LA diameter (3.8 ± 0.4 cm vs. 3.8 ± 0.4 cm; *p* = 1.00), AF duration (14.3 ± 5.4 months vs. 13.6 ± 5.3 months; *p* = .43) between the STAR mapping and conventional ablation cohort. The proportion of patients with structural heart disease was also not significantly different (*p* = 1.00).

**Table 1:** Baseline characteristics

**Baseline characteristics**	**STAR Cohort *n* = 65 **		Propensity Cohort *n* = 65	***p*‐value**
Age, years mean ± *SD*	60.9 ± 9.4		64.1 ± 10.7	.19
Male, *n* (%)	47 (72.3)		45 (69.2)	.85
Antiarrhythmic drugs, *n* (%)	46 (70.8)		44 (67.7)	.85
Hypertension, *n* (%)	16 (24.6)		18 (27.7)	.84
Diabetes mellitus, *n* (%)	0 (0)		0 (0)	1.00
TIA/CVA *n* (%)	2 (3.1)		0 (0)	.50
Structural heart disease, *n* (%)	4 (6.2)		5 (7.7)	1.00
Previous cardiac surgery, *n* (%)	1 (1.5)		3 (4.6)	.62
LA diameter, (cm) mean ± *SD*	3.8 ± 0.4		3.8 ± 0.4	1.00
AF duration, months mean ± *SD*	14.3 ± 5.4		13.6 ± 5.3	.43

Abbreviations: AF, atrial fibrillation; LA, left atrium; STAR, Stochastic Trajectory Analysis of Ranked signal; TIA/CVA: transient ischemic event/cerebrovascular accident.

#### 3.1.2 Procedural data

The procedure times were not significantly different in the STAR mapping cohort compared with the conventional ablation cohort (225.4 ± 65.6 min vs. 219.0 ± 64.8 min; *p* = .74). The total procedure radiofrequency time was not significantly higher in the STAR mapping cohort compared with the conventional ablation cohort (63.5 ± 10.2 vs. 62.4 ± 15.5; *p* = .63).

In the STAR mapping cohort one patient experienced cardiac tamponade which was noted at the end of the procedure which required pericardiocentesis. No other complications were encountered in this cohort. In the propensity‐matched cohort five patients experienced a procedural complication which included four cardiac tamponades that required pericardiocentesis and one pseudoaneurysm that required thrombin injection.

#### 3.1.3 Procedural electrophysiological and follow‐up endpoints

In the conventional ablation cohort a majority of patients had linear ablation (51/65, 78.5%; Table 2) of which roof line ablation was most frequent (26/51, 51.0%). Thirty‐eight out of the 65 patients underwent CFAE ablation (58.5%). Twenty‐four out of the 65 patients (36.9%) underwent both linear and CFAE ablation (Table S2).

**Table 2:** Procedural and follow‐up differences between STAR mapping and propensity matched cohort

	**STAR Cohort *n* = 65**	**Propensity Cohort *n* = 65**	***p*‐value**
Procedural data			
General anesthetic for procedure	18 (27.7)	16 (24.6)	.84
AF termination with ablation	45 (69.2)	10 (15.4)	<.001
Procedural duration, min mean ± *SD*	225.4 ± 65.6	231.3 ± 52.7	.63
Total ablation, min mean ± *SD*	63.5 ± 10.2	62.4 ± 15.5	.03
Fluoroscopy time, min mean ± *SD*	1.9 ± 3.2	4.8 ± 6.4	.36
Complications, *n* (%)	1 (1.5)	5 (7.7)	.21
Line ablation *n* (%)		51 (78.5)	
Roof	/	26 (51.0)	
Mitral	/	18 (35.3)	
CTI	/	17 (33.3)	
Septal	/	4 (7.8)	
Follow‐up data			
Follow‐up, months mean ± *SD*	29.5 ± 3.7	20.5 ± 8.1	<.001
Freedom from AF/AT, *n* (%)	52 (80.0)	32 (49.2)	<.001
Breakdown of follow‐up data			
Single procedure, *n* (%)	52 (80.0)	32 (49.2)	<.001
Two procedures, *n* (%)	11 (16.9)	28 (43.1)	.002
Three procedures, *n* (%)	2 (3.1)	5 (7.7)	.44
Mechanism of arrhythmia that recurred after first procedure	4 AF 9 AT	26 AF 7 AT	<.001 .79
			

Abbreviations: AF, atrial fibrillation; AT, atrial tachycardia; STAR, Stochastic Trajectory Analysis of Ranked signals.

AF termination was achieved with 51 (33 to AT and 18 to sinus rhythm) out of the 65 patients in the STAR mapping cohort (Figures [Link], [Link], [Link]; 78.5%) versus 10 out of the 65 patients in the conventional ablation cohort (15.4%; *p* < .001). In both cohorts the AT were mapped and ablated to achieve sinus rhythm at the end of the procedure in all except two patients. The patients who remained in AF all underwent DC cardioversion to sinus rhythm.

**Figure 3:**
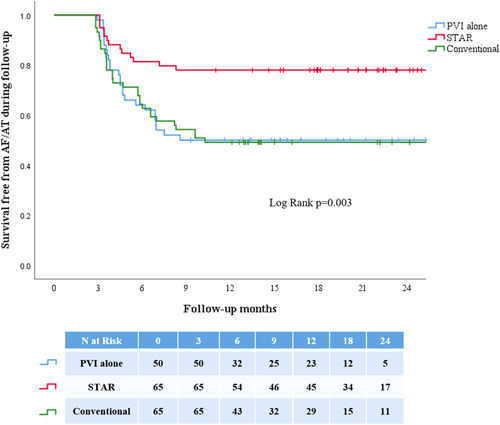
Demonstrates Kaplan–Meier curves that compares survival free from AF/AT during follow‐up in the STAR mapping cohort and that achieved in the conventional ablation cohort and PVI alone ablation cohort. The survival free rates were significantly higher in the STAR mapping cohort than the two other cohorts. A majority of the patients with arrhythmia recurrence presented at 3 months follow‐up. AF, atrial fibrillation; AT, atrial tachycardia; PVI, pulmonary vein isolation; STAR, Stochastic Trajectory Analysis of Ranked signals

The average follow‐up was longer in the STAR cohort (29.5 ± 3.7 months vs. 20.5 ± 8.1 months; *p* < .001). Recurrence of AF/AT after a single procedure occurred in 13/65 (20.0%) patients in the STAR cohort compared with 33/65 patients (50.8%) in the conventional ablation cohort (*p* < .001). Rates of recurrent AF were 4/13 patients (30.8%) in the STAR cohort compared with 26/33 patients (78.8%) in the conventional ablation cohort (*p* = .005). Of the four patients in STAR with AF all presented with persistent AF. In the conventional cohort 21 out of the 26 patients presented in persistent AF while the remaining were paroxysmal (4/4 vs. 21/26; *p* = 1.00). There was no excess of AT in the STAR cohort compared with the conventional ablation cohort (9/65 vs. 7/65; *p* = .79). The survival freedom from AF/AT was significantly higher in the STAR mapping cohort than the conventional ablation cohort (Figure 3).

In the STAR mapping cohort 52 patients underwent one procedure, 11 underwent two procedures, and 2 underwent three procedures. Out of the 13 patients that had arrhythmia recurrence during follow‐up, eight patients underwent one procedure for AT, one patient underwent two procedures for AT, and four patients underwent one procedure for AF of which one also had a second one for AT. In the the conventional ablation cohort 32 patients underwent one procedure, 28 patients had two procedures, and five patients had three procedures. The number of patients undergoing a repeat procedure during follow‐up was therefore lower in the STAR mapping cohort (13 vs. 33; *p* < .001). The average number of procedures in the STAR mapping cohort was also significantly lower than the conventional ablation cohort (1.3 ± 0.5 in the STAR mapping cohort vs. 1.6 ± 0.6 conventional ablation cohort; *p* = .001).

All patients in the STAR mapping cohort had antiarrhythmic drugs stopped at follow‐up. In the conventional ablation cohort 16 (24.6%) patients remained on an antiarrhythmic drugs during follow‐up (*p* < .001).

### 3.2 PVI plus STAR mapping guided ablation versus PVI alone ablation

#### 3.2.1 Baseline characteristics

Baseline characteristics are shown in Table 3. Fifty patients underwent planned PVI alone ablation during the same period. There was no significant difference in the use of antiarrhythmic drugs (46/65 vs. 35/50; *p* = 1.00), LA diameter (3.8 ± 0.4 cm vs. 3.9 ± 0.8 cm; *p* = .43), AF duration (14.3 ± 5.4 months vs. 13.2 ± 4.3 months; *p* = .35) between the STAR mapping and PVI alone ablation cohort (Table 3).

**Table 3:** Differences between STAR mapping and PVI alone ablation Cohorts

Baseline characteristics	STAR Cohort *n* = 59	PVI alone ablation Cohort *n* = 50	*p*‐value
Age, years mean ± *SD*	60.9 ± 9.4	61.3 ± 8.7	.39
Male, *n* (%)	47 (72.3)	38 (76.0)	.68
Antiarrhythmic drugs	46 (70.8)	35 (70.0)	1.00
Hypertension, *n* (%)	16 (24.6)	13 (26.0)	1.00
Diabetes mellitus, *n* (%)	0	0	1.00
TIA/CVA, *n* (%)	2 (3.1)	0	1.00
Structural heart disease, *n* (%)	4 (6.2)	3 (6.0)	.47
Previous cardiac surgery, *n* (%)	1 (1.5)	1 (2.0)	1.00
LA diameter, cm mean ± *SD*	3.8 ± 0.4	3.9 ± 0.8	.43
AF duration months, mean ± *SD*	14.3 ± 5.4	13.2 ± 4.3	.35
Procedural related data			
General anesthetic for procedure	18 (27.7)	14 (21.5)	1.00
AF termination with ablation	45 (69.2)	13 (26.0)	<.001
Procedural duration min ± *SD*	225.4 ± 65.6	208.5 ± 59.4	.17
Total ablation, min ± *SD*	63.5 ± 10.2	40.2 ± 19.5	<.001
Fluoroscopy time, min ± *SD*	1.9 ± 3.2	5.7 ± 4.9	<.001
Complications, *n* (%)	1 Tamponade (1.5)	1 Tamponade (1.7)	1.00
Follow‐up related data			
Follow‐up months, mean ± *SD*	29.5 ± 3.7	22.6 ± 5.8	<.001
Freedom from AF/AT, *n* (%)	52 (80.0)	25 (50.0)	<.001

Abbreviations: AF, atrial fibrillation; AT, atrial tachycardia; LA, left atrium; PVI, pulmonary vein isolation; STAR, Stochastic Trajectory Analysis of Ranked signals; TIA/CVA: transient ischemic event/cerebrovascular accident.

#### 3.2.2 Procedural data

The procedure times were not significantly different in the STAR mapping cohort compared with the PVI alone ablation cohort (225.4 ± 65.6 min vs. 208.5 ± 59.4 min; *p* = .17; Table 3). The total procedure radiofrequency time was significantly higher in the STAR mapping cohort compared to the PVI alone ablation cohort (63.5 ± 10.2 min vs. 40.2 ± 19.5 min; *p* < .001). As in the STAR mapping cohort, one patient experienced cardiac tamponade requiring pericardiocentesis in the PVI alone ablation cohort.

#### 3.2.3 Procedural electrophysiological and follow‐up endpoints

These endpoints are summarized in Table 3. A larger proportion of patients in the STAR guided ablation cohort achieved AF termination with ablation compared with the PVI alone ablation cohort (*n* = 51, 78.5% vs. *n* = 13, 26.0%; *p* < .001).

The average follow‐up was significantly longer in the STAR cohort (29.5 ± 3.7 months vs. 22.6 ± 5.8 months; *p* < .001). Recurrence of arrhythmia was significantly higher in the PVI alone ablation cohort compared with the STAR cohort (25/50, 50% vs. 13/65, 20.0%; *p* = .001). Rates of recurrent AF were 4/65 (6.2%) in the STAR cohort compared with 22/50 (44.0%) in the PVI alone ablation cohort (*p* < .001). Of the four patients in STAR with AF all presented with persistent AF. In the PVI alone cohort 19 out of the 22 patients presented in persistent AF while the remaining were paroxysmal (4/4 vs. 19/22; *p* = 1.00). There was no excess of AT in the STAR cohort compared with the PVI alone cohort (9/65 vs. 3/50; *p* = .23). The survival free from arrhythmia on Kaplan–Meier analysis was significantly higher in the STAR mapping cohort than the PVI alone ablation cohort (Figure 3).

In the PVI alone ablation cohort all 25 patients with arrhythmia recurrence underwent a secocnd procedure. The number of patients undergoing a repeat procedure was therefore significantly lower in the STAR mapping cohort (13/65 vs. 25/50; *p* = .003). Further to this, 10 patients underwent three procedures in the PVI alone ablation cohort (20.0%) versus two in the STAR mapping cohort (3.1%; *p* = .005). The average number of procedures was significantly higher in the PVI alone ablation cohort compared with the STAR mapping cohort (1.7 ± 0.8 PVI alone ablation cohort vs. 1.3 ± 0.5 in the STAR mapping cohort; *p* < .001). In the PVI alone ablation cohort 16 (27.1%) patients remained on anti‐arrythmic drugs versus no patients in the STAR mapping cohort (*p* = .001).

## 4 DISCUSSION

These data suggest that ablation guided by STAR mapping in addition to PVI is associated with superior outcomes compared with PVI alone or conventional ablation incorporating PVI with either CFAEs and/or linear ablation in a propensity matched cohort. Patients undergoing ablation guided by STAR mapping using either global mapping with basket catheters or sequential mapping with PentaRay catheter were more likely to achieve AF termination as a procedural endpoint and with significantly higher rates of freedom from AF/AT during long‐term follow‐up than either the PVI cohort or the propensity matched cohort of conventional ablation. The rates of AF recurrence in the STAR guided ablation cohort were markedly lower than the other two cohorts, without any excess of AT recurrence compared with conventional ablation or PVI alone ablation. The STAR guided ablation cohort underwent significantly fewer procedures than either of the other two cohorts. Further to this, in the STAR mapping cohort the antiarrhythmic drugs were stopped in all patients while in the PVI alone and conventional ablation cohort approximately 30% of patients remained on an antiarrhythmic drugs.

All patients in the STAR mapping cohort underwent Holter monitoring at 6 and 12 months. In the conventional ablation cohort and PVI alone ablation cohort the patients did not undergo systematic Holter monitoring and the decision for Holter monitoring was based on symptoms and physicians discretion. Therefore, it is possible that the arrhythmia recurrence rate was underestimated in these cohorts. Further to this, all patients in the STAR mapping cohort had the antiarrhythmic drugs stopped at 3 months as per study protocol whilst this was not the case in the two other cohorts, which may also have reduced the AF/AT recurrence rates in the other two cohorts. Even though all patients in both cohorts underwent a minimum of 12 months follow‐up, the follow‐up duration was also significantly longer in the STAR mapping cohort compared to the other two cohorts. Nevertheless, despite these disadvantages, there was still a clear difference in outcomes favouring the STAR guided ablation cohort.

The freedom from AF/AT rates in the conventional ablation cohort and PVI ablation only cohort is compatible to that reported in previous studies. The STAR AF2 trial concluded that PVI with either CFAE or/and linear ablation was not superior to a PVI only ablation approach.^1^ The findings from this study further supports this. STAR mapping guided ablation was superior to either of these ablation approaches. Further to this, a majority of the patients included in this study had an AF duration of more than 12 months making them predominantly long‐standing persistent AF patients. The results in the other two cohorts are perhaps as expected from the literature for long standing persistent AF. The outcomes in the STAR guided ablation cohort suggest that the utility of this approach is not restricted to early persistent AF, and still achieves good procedural end‐points and clinical outcomes in those with more advanced disease. Furthermore, the STAR guided approach to ablation did not result in significantly longer procedure times and had an ablation duration comparable with the conventional ablation cohort.

In the STAR guided ablation cohort only 4/65 patient (6.2%) had recurrent AF compared with 26/65 (40.0%) in the conventional ablation cohort and 22/50 (44.0%) in the PVI alone cohort, without any excess of AT. These data arguably lend weight to the driver hypothesis for maintenance of AF. The rationale for ablation at sites where wavefronts originate is that localized sources maintain AF. However, STAR mapping does not differentiate between mechanisms at the leading site and simply identifies the leading areas.

It has long been suspected that further ablation in the form of CFAE ablation and linear ablation might reduce the rate of AF recurrence at the expense of more AT recurrence. However, with targeted ablation using STAR mapping there was no excess of AT recurrence in the STAR mapping cohort. Consequently, fewer patients required a repeat procedure in the STAR guided ablation cohort, and the average number of procedures was fewer. If this increase in long‐term single procedure success and reduction in the need for repeat procedures is borne out in a larger multi‐center experience this would have substantial economic implications in terms of cost efficacy for persistent AF ablation.

The propensity matching was performed including all major variables that have been shown to influence arrhythmia recurrence post catheter ablation. This allowed the establishment of a cohort that allowed a reliable comparison with the STAR mapping cohort in regard to procedural and follow‐up endpoints, and indeed the outcomes in this cohort were much as expected from other series and trials in the literature. The superiority of the STAR mapping guided ablation needs to be further evaluated in multicenter randomized controlled trials such as the randomized Controlled Trial of STAR Mapping™ Guided Ablation for AF (ROC‐STAR) trial currently planned (NCT04442113).

### 4.1 Limitations

This study has several important limitations. First, this is a nonrandomized study. The STAR mapping cohort consists of a relatively small number of patients. However, this cohort of 65 patients was thought reasonable as a feasibility study and to give an impression of outcomes. The number of patients undergoing persistent AF ablation is similar to previously reported studies using a novel mapping system.^2^, ^3^ The number of consecutive patients undergoing PVI alone ablation during the same time period as the STAR mapping patients were smaller making propensity matching unfeasible for this cohort. This has resulted in some heterogeneity in methods, in that the conventional ablation cohort were propensity matched to the STAR mapping cohort, whereas the PVI alone ablation cohort consisted of a smaller number of consecutive patients. However, baseline characteristics known to impact AF procedural outcomes in the PVI alone ablation and STAR mapping cohorts were not significantly different. Furthermore, the clinical outcomes in the PVI alone ablation cohort and the conventional ablation cohort are very much in line with the published literature, so it seems this heterogeneity in methods has not distorted the clinical outcomes and the comparisons should remain valid. All procedures included in this study were performed in a single center; it is therefore important to evaluate the efficacy of the STAR mapping method in other centers to ensure the findings are reproducible by others. However, as the cases in the conventional ablation cohort and PVI alone ablation cohort were performed in the same center these cohorts ought still to be comparable. All procedures were performed by senior electrophysiologists. There was overlap in the operators performing ablations in the different groups, but we recognize that differences in operators between groups may cause further confounding in this non‐randomized comparison. A multicenter study is being planned to assess the efficacy of PVI plus STAR guided ablation compared to PVI alone ablation in a randomized controlled trial powered to assess clinical outcomes (NCT04442113).

## 5 CONCLUSIONS

Ablation guided by STAR mapping in addition to PVI was associated with a higher rate of AF termination and freedom from AF/AT during long‐term follow‐up than PVI alone ablation or conventional ablation strategy incorporating CFAE and/or linear ablation in this nonrandomized comparison. This supports the driver hypothesis for maintenance of AF and suggests an ablation strategy targeting drivers in this way may be useful in the treatment of AF. This was achieved without any significant difference in procedure time or any difference in radiofrequency ablation time compared to conventional ablation. STAR mapping guided ablation was not associated with any excess of ATs and was associated with fewer patients undergoing repeat procedures, a lower average number of procedures, and less use of antiarrhythmic drugs compared with the other two cohorts. The efficacy of ablation guided by STAR mapping needs to be further evaluated in a multicenter randomized controlled trial.

## Supporting information

Supporting information.

Supporting information.

Supporting information.

Supporting information.

Supporting information.

Supporting information.

## Data Availability

The data that support the findings of this study are available from the corresponding author upon reasonable request.
