# Supplementary figures and images for: Ablation guided by STAR‐mapping in addition to pulmonary vein isolation is superior to pulmonary vein isolation alone or in combination with CFAE/linear ablation for persistent AF
Source: J Cardiovasc Electrophysiol. 2021 Jan 9;32(2):200–9. doi: 10.1111/jce.14856 (PMC8607469; doi:10.1111/jce.14856)

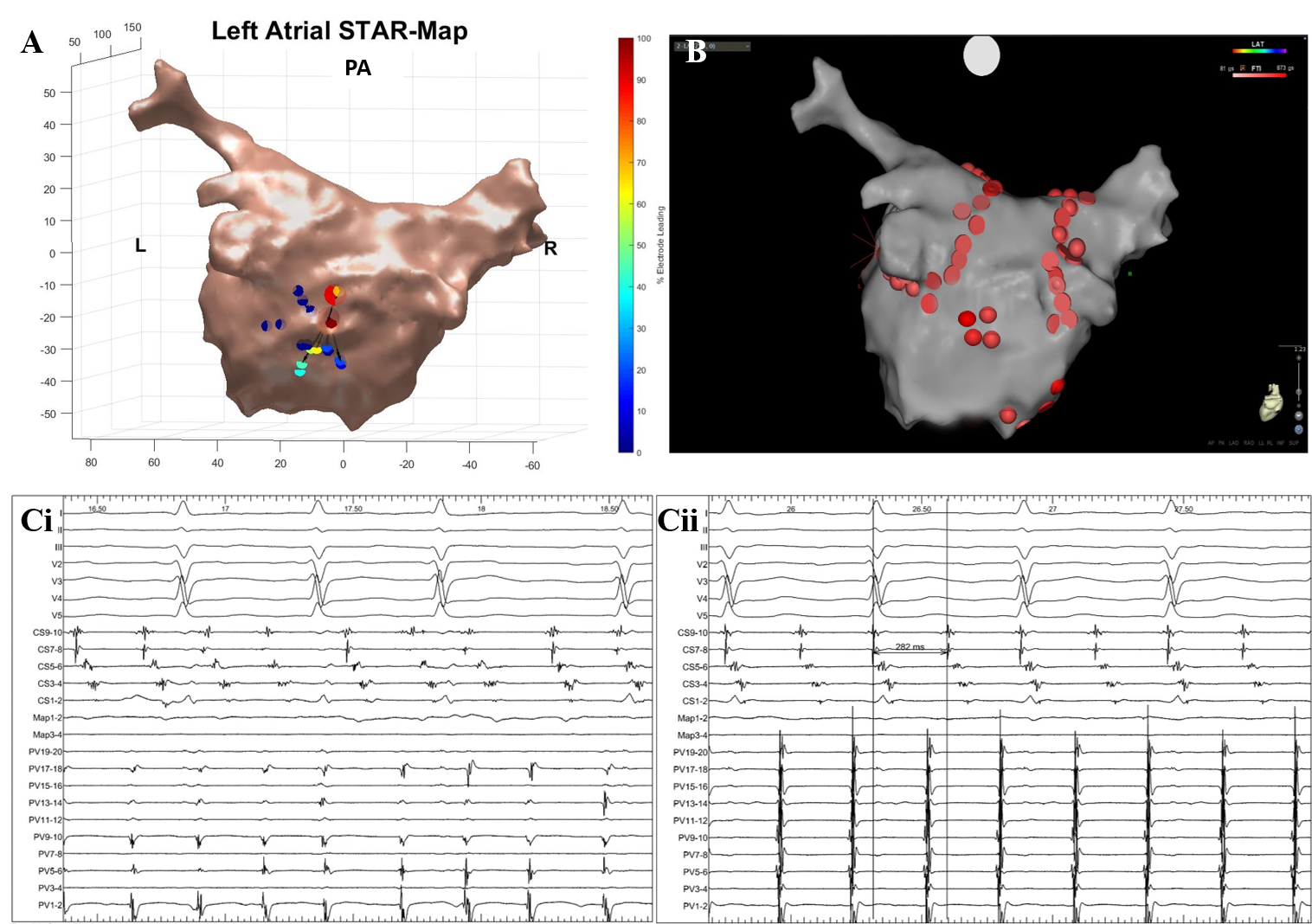

Supplement: Supplementary file 1 — Supporting information. [file JCE-32-200-s005.tif]

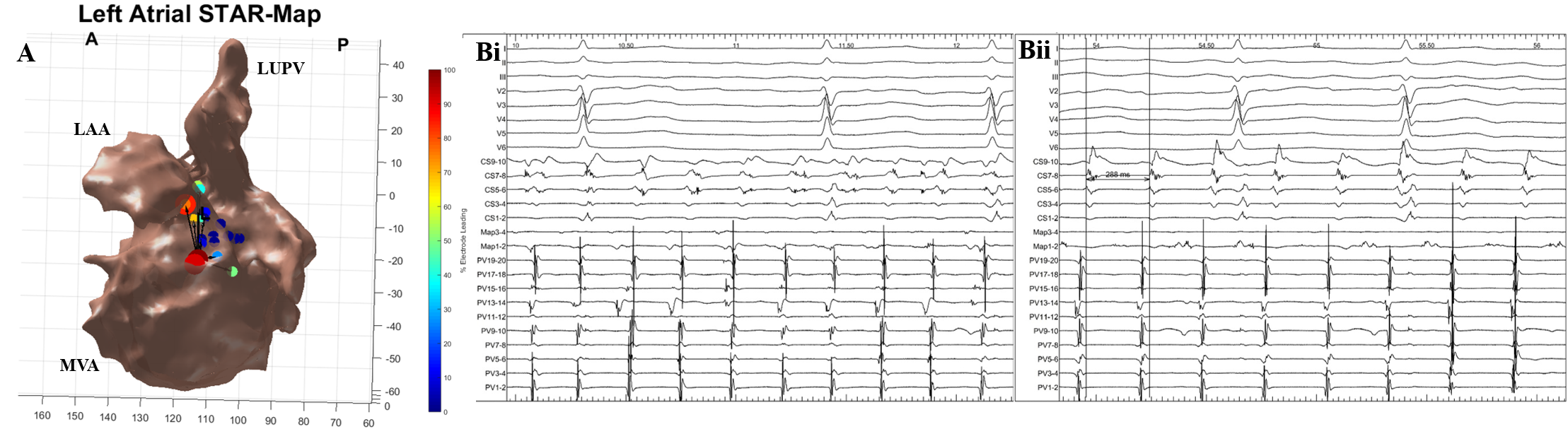

Supplement: Supplementary file 2 — Supporting information. [file JCE-32-200-s004.tif]

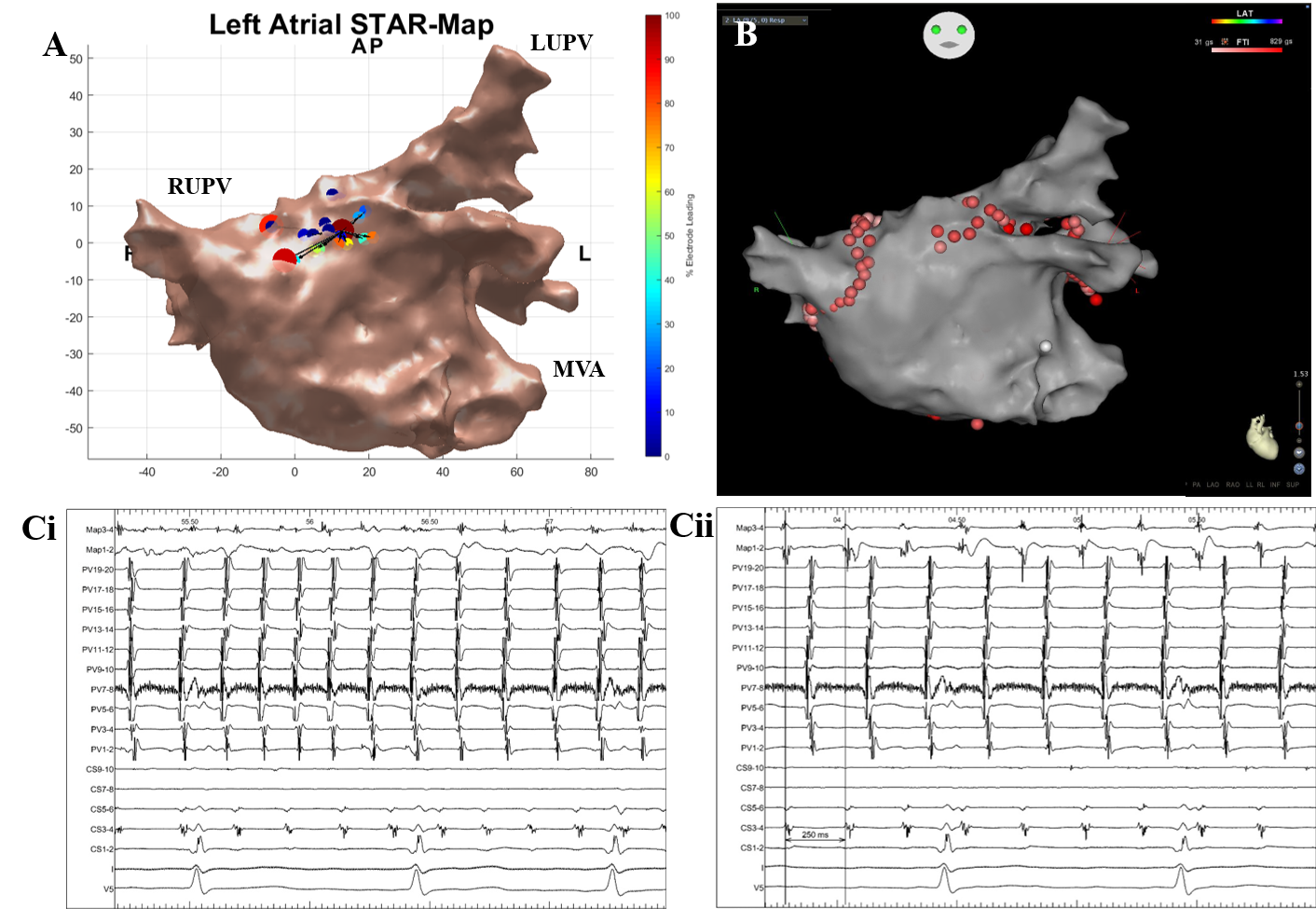

Supplement: Supplementary file 3 — Supporting information. [file JCE-32-200-s006.tif]
