# Supplementary material for: Ablation guided by STAR‐mapping in addition to pulmonary vein isolation is superior to pulmonary vein isolation alone or in combination with CFAE/linear ablation for persistent AF
Source: J Cardiovasc Electrophysiol. 2021 Jan 9;32(2):200–9. doi: 10.1111/jce.14856 (PMC8607469; doi:10.1111/jce.14856)
Supplement: Supplementary file 4 — Supporting information. [file JCE-32-200-s001.docx]

**SUPPLEMENTAL FIGURE LEGEND**

***Supplemental Figure 1A-C-*** Demonstrates ***A)*** a STAR map of the LA in a posterior-anterior view that shows an AFD on the posterior-inferior wall. ***B)*** where 2.9 minutes of ablation as shown on the CARTO LA geometry resulted in ***Ci-ii)*** organization of AF to AT as shown on the electrograms obtained from BARD. STAR map guided posteroseptal ablation as shown on the CARTO map did not result in a study-defined ablation response.

LUPV- Left upper pulmonary vein

RUPV- Right upper pulmonary vein

***Supplemental Figure 2A-C-*** Demonstrates ***A)*** a STAR map of the LA in a lateral view that shows an AFD mid lateral wall where 2.5 minutes of ablation resulted in ***Bi-ii)*** organization of AF to AT as shown on the electrograms obtained from BARD.

LUPV- Left upper pulmonary vein

LAA- Left atrial appendage

MVA- Mitral valve annulus

***Supplemental Figure 3A-C-*** Demonstrates ***A)*** a STAR map of the LA in a titled anterior-posterior view that shows an AFD roof/high anterior wall ***B)*** where 2.2 minutes of ablation as shown on the CARTO LA geometry resulted in ***Ci-ii)*** organization of AF to AT as shown on the electrograms obtained from BARD. STAR map guided septal ablation as shown on the CARTO map resulted in cycle length slowing of ≥30ms.

LUPV- Left upper pulmonary vein

RUPV- Right upper pulmonary vein

MVA- Mitral valve annulus
