# Supplementary material for: Ablation guided by STAR‐mapping in addition to pulmonary vein isolation is superior to pulmonary vein isolation alone or in combination with CFAE/linear ablation for persistent AF
Source: J Cardiovasc Electrophysiol. 2021 Jan 9;32(2):200–9. doi: 10.1111/jce.14856 (PMC8607469; doi:10.1111/jce.14856)
Supplement: Supplementary file 5 — Supporting information. [file JCE-32-200-s002.docx]

***Supplemental Table 1-*** *The variables used in the propensity matching.*

| Age  Male  Anti-arrhythmic drugs  Hypertension  Diabetes mellitus  Transient ischaemic attack/Cerebrovascular attack  Structural heart disease  Previous cardiac surgery  Left atrial diameter |
| --- |
| AF duration |
