# Supplementary material for: Ablation guided by STAR‐mapping in addition to pulmonary vein isolation is superior to pulmonary vein isolation alone or in combination with CFAE/linear ablation for persistent AF
Source: J Cardiovasc Electrophysiol. 2021 Jan 9;32(2):200–9. doi: 10.1111/jce.14856 (PMC8607469; doi:10.1111/jce.14856)
Supplement: Supplementary file 6 — Supporting information. [file JCE-32-200-s003.docx]

**Supplemental Table 2-** Demonstrates the anatomical site of ablation in the STAR mapping cohort and the type of ablation in the conventional ablation cohort.

| **Patient ID** | | **STAR Cohort n=65** | **Conventional Ablation Cohort**  **n=65** |
| --- | --- | --- | --- |
| 1 | PVI^*^  Low anterior  High anterior  Endocardial proximal CS^Ͳ^ | | PVI  CFAE^Ŧ^ |
| 2 | PVI  Roof/LAA^§^  Mid anterior | | PVI  Roof line |
| 3 | PVI  Mid roof  Mid posterior  Low anterior  Septal RA^ǀǀ^ | | PVI  Mitral line  Roof line |
| 4 | PVI  Mid roof  Mid posterior  Low anterior  Lateral RA | | PVI  CTI^♯^ line |
| 5 | PVI  Lateral inferior  High anterior | | PVI  CFAE  CTI line |
| 6 | PVI  Low anterior  High anterior/Mid roof | | PVI  CTI line |
| 7 | PVI  Posterior septal  Anterior LAA | | PVI  Roof line |
| 8 | PVI  Anterior LAA  Septal RA | | PVI  Roof line |
| 9 | PVI  Low anterior  High lateral | | PVI  Roof line  CFAE |
| 10 | PVI  Roof/LAA  Lateral | | PVI  Roof line  Mitral line  CTI line |
| 11 | PVI  Mid anterior  Roof | | PVI  CFAE  Roof line |
| 12 | PVI  Lateral  Inferior RLPV  Mid posterior | | PVI  CFAE |
| 13 | PVI  LAA/Roof  Inferior | | PVI  CFAE |
| 14 | PVI  High anterior  Mid posterior  Roof | | PVI  CFAE |
| 15 | PVI  Roof/LAA  Inferior to RUPV  Posterior-inferior | | PVI  CFAE  Mitral line |
| 16 | PVI  High anterior  Posterolateral  Lateral | | PVI  Roof line  CTI line |
| 17 | PVI  LAA  Distal endocardial CS  LAA/Roof | | PVI  Roof line  CFAE |
| 18 | PVI  Lateral  Anteroseptum | | PVI  Roof line  CTI line  Septal line  CFAE |
| 19 | PVI  Inferolateral  Roof  Mid anterior | | PVI  CFAE |
| 20 | PVI  Lateral  Roof | | PVI  CFAE  Roof line  Mitral line |
| 21 | PVI  Anteroseptum | | PVI  CFAE |
| 22 | PVI  Anterior LAA  Anteroseptum  Posterolateral | | PVI  Roof line  Mitral line  CFAE |
| 23 | PVI  Mid roof  Anteroseptum  Mid posterior | | PVI  CFAE  Roof line  Mitral line |
| 24 | PVI  Mid posterior  Endocardial distal CS  Mid roof | | PVI  CFAE |
| 25 | PVI  Mid anterior  Roof  Mid posterior  Anteroseptum | | PVI  CFAE |
| 26 | PVI  Endocardial proximal CS  High posterior  Anterior roof  High anterior | | PVI  Roof line |
| 27 | PVI  High posterior  Anteroseptum | | PVI  CFAE  Mitral line |
| 28 | PVI  Lateral  Posteroseptal  Mid anterior | | PVI  Roof line |
| 29 | PVI  Inferior RUPV  Mid lateral  Mid posterior | | PVI  Roof line |
| 30 | PVI  Roof  Mid anterior | | PVI  Mitral line  Roof line |
| 31 | PVI  LAA/Roof  Low anterior  Mid lateral | | PVI  CTI line |
| 32 | PVI  Roof  Mid anterior  Mid lateral | | PVI  CTI line |
| 33 | PVI | | PVI  Roof line  CTI line |
| 34 | PVI | | PVI  Mitral line  CFAE |
| 35 | PVI | | PVI  Roof line  Septal line |
| 36 | PVI  Lateral  Anterior/Mid septal  Anterior LAA  Mid posterior  Lateral | | PVI  CFAE |
| 37 | Mid posterior  Mid roof  LAA ridge/lateral | | PVI  Mitral line |
| 38 | Mid anterior | | PVI  CTI line |
| 39 | PVI | | PVI  CFAE |
| 40 | LAA/Roof  Anterior | | PVI  Mitral line |
| 41 | LAA/Roof  Mid anterior | | PVI  CFAE |
| 42 | High anterior  Posterior roof  Endocardial proximal CS | | PVI  CFAE  CTI line |
| 43 | Anterior RUPV^×^  Roof/RUPV | | PVI  CTI line |
| 44 | Mid anterior  Mid roof | | PVI  CFAE  CTI line |
| 45 | PVI | | PVI  CFAE |
| 46 | Septal  Roof | | PVI  Mitral line |
| 47 | Mid lateral  Posteroseptal  Low posterior | | PVI  Roof line  CFAE |
| 48 | Low septal  Mid lateral | | PVI  Septal line |
| 49 | Low septum  Low anterior  Posterior/Lateral | | PVI  CFAE |
| 50 | Mid roof  Lateral | | PVI  CTI line |
| 51 | High anterior  Mid roof  Inferior/Lateral | | PVI  Roof line  Mitral line |
| 52 | Roof/LAA  Mid posterior  Low septum  Mid roof  Mid anterior  Anteroseptum | | PVI  CFAE  CTI line |
| 53 | High anterior  Roof/RUPV  Roof/LAA | | PVI  CFAE  Roof line |
| 54 | Mid posterior  High anterior  Mid roof | | PVI  Mitral line  CFAE |
| 55 | Posterior/Inferior  LAA/Roof  Low anterior  Mid lateral | | PVI  Mitral line  CFAE  CTI line |
| 56 | High posterior  Mid anterior | | PVI  Septal line  CFAE |
| 57 | Lateral  LAA/Roof  RUPV/Anteroseptum  Mid septum | | PVI  CFAE  CTI line |
| 58 | High posterior  Mid anterior | | PVI  CFAE |
| 59 | Mid anterior Lateral | | PVI  Mitral line |
| 60 | Mid posterior  Mid roof | | PVI  Roof line |
| 61 | Mid anterior  Inferior RUPV  Septum | | PVI  Roof line  CFAE |
| 62 | Posterior/inferior  Distal endocardial CS | | PVI  Roof line  CFAE |
| 63 | Roof/LAA  Low anterior | | PVI  Roof line |
| 64 | Mid lateral  Posteroseptum | | PVI  Mitral line  CFAE |
| 65 | AT on PVI | | PVI  Mitral line  CFAE |

^*^PVI- Pulmonary vein isolation

^Ͳ^CS- Coronary sinus

^Ŧ^CFAE- Complex fractionated atrial electrograms

^§^LAA- Left atrial appendage

^ǀǀ^RA- Right atrium

^♯^CTI- Cavo-tricuspid isthmus

^×^RUPV- Right upper pulmonary vein
